# A multimodal benchmark dataset for evaluating large language models on traditional Chinese opera understanding

**DOI:** 10.1038/s41597-026-07611-7

**Published:** 2026-07-01

**Authors:** Gengxian Cao, Shuo Hou, Ye Yang, Ruosong Liu, Hong Duan, Donghe Li

**Affiliations:** 1 The Music Education Center at the School of Humanities, Xi’an Jiaotong University, Xi’an, China; 2 School of Automation Science and Engineering, Xi’an Jiaotong University, Xi’an, China; 3 Shanghai DianJi University, Shanghai, China; 4 The Shaanxi Provincial Opera Research Institute, Xi’an, China

**Keywords:** Interdisciplinary studies, Arts, Scientific data, Databases, Arts

## Abstract

Benchmarking is essential for evaluating the capabilities of large language models (LLMs). However, existing multimodal benchmarks lack dedicated resources for traditional Chinese opera, a domain rich in cultural and visual complexity. To address this gap, we introduce the TCO-Dataset, a bilingual multimodal dataset designed to assess LLMs’ ability to interpret and reason about Chinese opera images. The dataset contains 1,000 multiple-choice questions paired with high-resolution images across eight major opera genres. Each sample includes a carefully selected image, a corresponding question focused on cultural and visual understanding, and an annotated answer for evaluation. The dataset supports both Chinese and English, enabling cross-lingual model assessment. All items were reviewed through multiple rounds of expert validation to ensure consistency and accuracy. The TCO-Dataset supports diverse applications, including still-image-based visual-cultural reasoning, cultural heritage preservation, and domain-specific AI development. Initial evaluations show significant performance variation across models, underscoring the dataset’s challenge and value for advancing multimodal understanding.

## Background & Summary

Large language models (LLMs) have driven substantial progress in natural language processing (NLP), achieving state-of-the-art results in tasks such as machine translation, open-ended text generation, and question answering^1–5^. Recent advancements in multimodal large language models (MLLMs), such as GPT-4^6^, Gemini^7^, Qwen-VL^8^, and InternVL^9^, have further extended these capabilities to vision-language tasks, enabling models to process and reason about image-text inputs jointly. This trend has created growing demand for rigorous and domain-sensitive evaluation benchmarks that can systematically assess LLM performance across diverse modalities and application contexts.

Benchmarking plays a central role in measuring model capabilities, exposing failure modes, and promoting progress in both general-purpose and domain-specific AI^10^. A number of vision-language benchmarks have been introduced to assess models’ ability to answer questions about everyday scenes (e.g. VQAv2^11^, GQA^12^), solve academic-style problems (e.g. ScienceQA^13^, MMMU^14^, MathVista^15^), and tackle multilingual or exam-style reasoning tasks (e.g. M3Exam^16^). While these datasets have significantly shaped the development of MLLMs, they are largely centered on factual, scientific, or commonsense reasoning and lack content that tests culturally grounded, fine-grained visual understanding^16–18^.

Traditional Chinese opera, a cornerstone of Chinese cultural heritage, represents a unique multimodal art form that integrates highly stylized visual, linguistic, and performative elements. Unlike conventional vision-language tasks, which predominantly focus on everyday object recognition and scene understanding^19^, opera understanding necessitates the integration of fine-grained visual perception and domain-specific knowledge. Key visual attributes—such as facial makeup, historically distinctive costumes, symbolic gestures, and stage settings—are deeply rooted in cultural and historical traditions, making them particularly challenging for models trained on mainstream multimodal datasets. Existing benchmarks, including ImageNet^20^ and Visual Genome^21^, primarily emphasize generic object classification and visual scene understanding, thus failing to address the complexities inherent in culturally specific visual reasoning. This gap underscores the necessity of a dedicated benchmark designed to assess LLMs’ capacity to process and interpret traditional Chinese opera images.

More recent benchmarks have made strides in broadening evaluation coverage, but still face limitations. ScienceQA^13^, while multimodal and covering 26 scientific domains, is strictly monolingual (English-only) and lacks artistic or symbolic content. MMMU^14^ and MathVista^15^ offer large-scale, multi-domain problems, yet the visual inputs are synthetic or diagrammatic, rather than grounded in real-world cultural media. M3Exam^16^ introduces multilingual multimodal testing across nine languages and several disciplines; however, its separation of visual and textual modalities, and absence of domain-specific cultural imagery, limit its capacity to assess deep visual-semantic alignment. These constraints illustrate a broader issue: current benchmarks are poorly equipped to evaluate MLLMs’ abilities in culturally embedded, symbolically rich tasks such as those found in traditional performance arts.

**Fig. 1:**
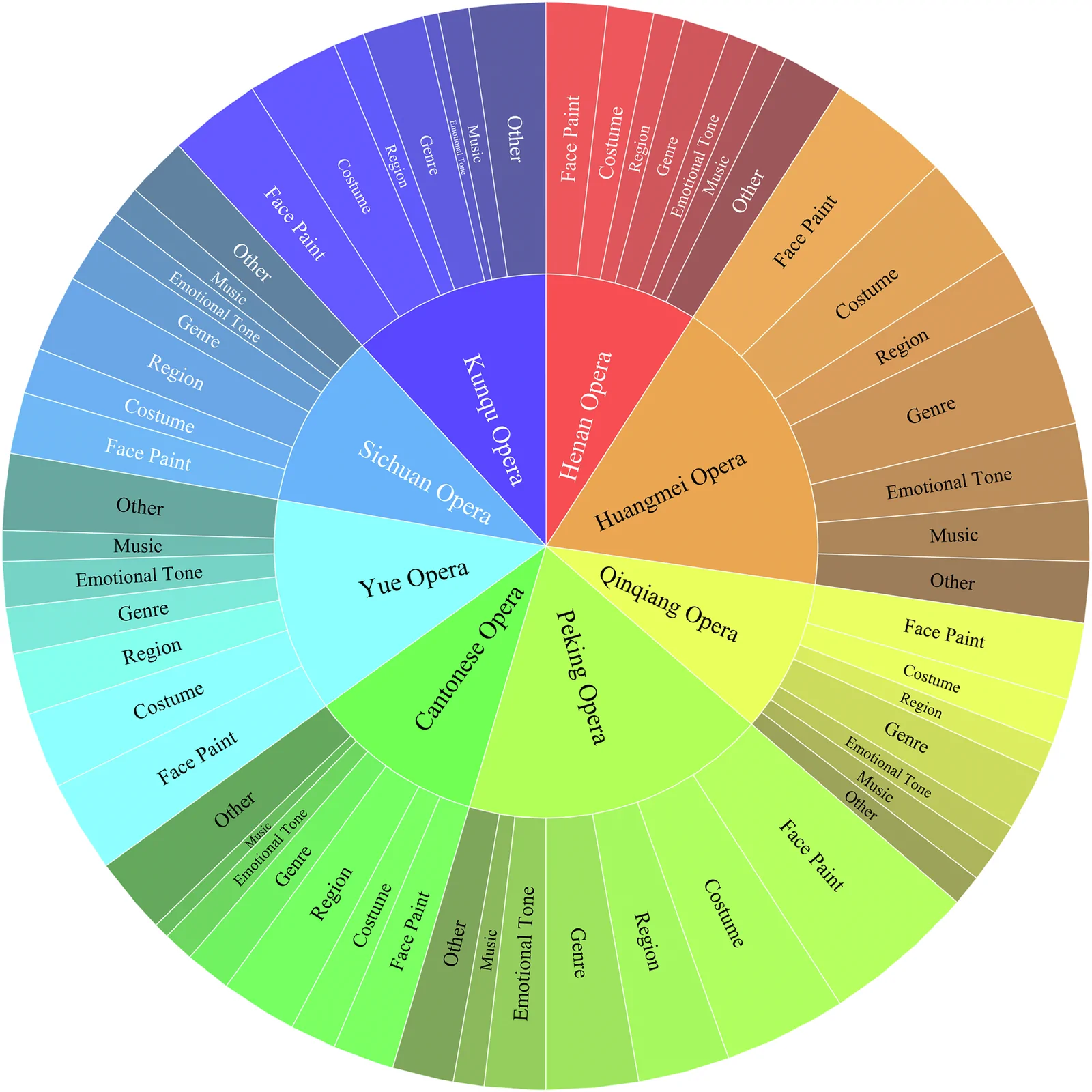
TCO-Dataset distribution.

To bridge this gap, we introduce TCO-Dataset, an opera-specific benchmark dataset developed for evaluating MLLMs on traditional Chinese opera still-image understanding. The data distribution is shown in Fig. 1. The dataset contains 1,000 samples spanning eight major opera genres, with each item organized as an opera still, a multiple-choice question, an answer label, and structured metadata. The detailed file organization and metadata schema are described in the Data Records section.

The questions span multiple categories, including opera genre classification, historical costume attribution, facial makeup symbolism, character role identification, prop recognition, and emotional tone analysis. In contrast to conventional benchmarks, TCO-Dataset features culturally dense visuals, domain-specific questions, and task formats that integrate visual detail with external cultural knowledge. Furthermore, all benchmark questions are available through Chinese and English metadata fields, while the original authored language distribution is documented separately to support controlled cross-lingual evaluation.

To ensure strict copyright compliance and data provenance, all images were curated from primary materials collected, digitized, or documented by the authors and their affiliated institution, including institutionally held digital records and on-site photography internally organized within the XJTU-ORD archival management system. Data annotation was carried out manually by domain experts—including scholars and professional practitioners of Chinese opera—following a multi-stage review protocol. Each item was subjected to three rounds of expert validation to ensure label accuracy, consistency across question types, and cultural fidelity. The dataset adheres to FAIR data principles, ensuring that it is findable, accessible, interoperable, and reusable for the broader research community.

This work makes the following contributions:

1. We present TCO-Dataset, a bilingual, fine-grained benchmark dataset specifically designed for evaluating MLLMs on traditional Chinese opera understanding. To the best of our knowledge, it is the first opera-specific bilingual benchmark of this kind for traditional Chinese opera still-image understanding.
2. We conduct a comprehensive evaluation of nine leading vision-language models on complex visual-cultural reasoning tasks, demonstrating persistent performance gaps.
3. We identify key limitations in current models’ ability to interpret cultural symbols, differentiate visually similar artistic styles, and contextualize historical knowledge, paving the way for improved model design.

TCO-Dataset offers a challenging, culturally grounded benchmark for advancing the development of multimodal models that can reason effectively over still images in high-context, domain-specific visual environments. It supports ongoing work in cross-lingual understanding, cultural AI, and domain adaptation, and is intended to foster more inclusive and representative benchmarking in the vision-language community.

## Methods

The TCO-Dataset was constructed through a multi-stage pipeline involving image collection, expert-guided annotation, structured data organization, and rigorous quality assurance. The entire process was designed to ensure that the dataset is culturally representative, linguistically diverse, technically consistent, and suitable for evaluating fine-grained multimodal reasoning in large language models.

### Data Collection

The TCO-Dataset is a primary benchmark dataset constructed from source materials collected, digitized, documented, and curated by the authors and their affiliated institution, Xi’an Jiaotong University. These source materials are internally organized within the XJTU Opera Research Database (XJTU-ORD), which serves as a local archival management system rather than an externally maintained third-party repository. To ensure a culturally representative and visually diverse sample set, the released benchmark was constructed from two internally curated source streams: field photography and institutionally held digital archival records.

The source materials were collected and curated by members of the research team and affiliated collaborators from Xi’an Jiaotong University and partner opera practitioners through two complementary channels: (1) field photography conducted during live performances, rehearsal documentation, and cultural-recording activities in provinces including Henan, Anhui, and Shaanxi; and (2) digitization and restoration of institutionally held historical performance records. The source archive is currently stored and managed by the School of Automation Science and Engineering at Xi’an Jiaotong University for research, preservation, and dataset curation purposes.

Overall, the source-material pool used for dataset curation comprised approximately 2,500 candidate visual records collected through field documentation and institutionally held archival materials, with geographic coverage including Henan, Anhui, and Shaanxi. The released benchmark was constructed by selecting 1,000 items from this broader source pool under the visual and cultural quality-control criteria described below.

A substantial portion of the images was selected from the institution’s digitized collection of historical performance records. These materials underwent a rigorous digitization and restoration process. Manual filtering was applied to eliminate blurry or redundant frames, with additional technical criteria including: (1) minimum resolution of 1280 × 720 pixels for digital records, (2) adequate contrast and brightness levels (histogram analysis ensuring no clipping in more than 5% of pixels), and (3) clear focus on central subjects with minimal motion blur. Images were also assessed for color accuracy and lighting consistency to ensure faithful representation of costume colors and makeup details. This subset also includes professionally photographed stage stills accompanied by validated metadata such as opera title, performance year, and character role, which were used to verify the annotation process.

To further enrich the dataset and ensure coverage of contemporary interpretations, we conducted field photography in collaboration with local opera troupes in provinces such as Henan, Anhui, and Shaanxi. Photographs were taken using ambient lighting under natural stage conditions to preserve the authenticity of costume color and texture, with additional care to avoid overexposure of reflective materials and underexposure of dark costume elements. Photography was conducted using standardized camera settings (ISO 800-1600, f/2.8-f/4.0) to maintain consistent image quality across different venues. All performers granted written consent, and the resulting images were released under research-use agreements. This effort contributed approximately 150 high-resolution images to the dataset.

**These field materials form part of the primary source collection curated by the authors and their affiliated institution**.

Across all sources, we applied a uniform selection process to identify the most semantically informative and visually clear images. Inclusion criteria required that each image depict a complete or near-complete view of one or more performers, with distinguishable visual features such as symbolic facial makeup, historically accurate costume, or context-specific stage props. Additional compositional requirements included: (1) performers occupying at least 30% of the image frame, (2) clear visibility of facial features and costume details without significant shadows or occlusion, and (3) stable, non-tilted camera angles that preserve the natural proportions of subjects. Images with ambiguous composition, incomplete costume elements, or non-performance settings (e.g., backstage or audience views) were excluded. From an initial pool of approximately 2,500 candidates, 1,000 images were selected for final inclusion after review by eight independent annotators.

### Annotation Procedure

Each instance in the TCO-Dataset—consisting of an image, a multiple-choice question, and an answer label—was created through a multi-stage annotation pipeline that emphasized cultural accuracy, linguistic clarity, and task diversity. The annotation team included 12 individuals with complementary expertise, including Chinese opera professors, performance studies researchers, regional troupe actors, and bilingual annotators trained in linguistics. This composition was intended to combine domain-specialist knowledge, practical stage experience, and cross-lingual annotation support throughout the question-construction and review process.

Questions were designed to assess models’ ability to perform fine-grained reasoning grounded in both visual features and domain knowledge. To support more fine-grained benchmark analysis, we organized the questions into three difficulty levels according to the cognitive demands required for correct answering. Basic-level questions focus primarily on direct visual recognition of salient elements, such as opera genre, costume features, visible props, or role appearance. Intermediate-level questions require the integration of visual evidence with commonly known domain conventions, such as matching makeup patterns or costume styles to role categories or cultural meanings. Advanced-level questions require multi-step reasoning that connects visual cues with deeper cultural, historical, or narrative knowledge, such as plot identification, symbolic interpretation, or inference about regional and stylistic traditions. Eight primary question types were defined: opera genre classification, historical costume attribution, character role identification (e.g., Sheng, Dan, Jing, Chou), facial makeup symbolism, stage prop and instrument recognition, plot/story identification, emotional tone inference, and regional distribution awareness. These question types were further reviewed and assigned to the three difficulty levels during dataset curation to enable more granular evaluation of model performance across skill levels.

Each question was authored based on a selected image, with four answer choices and one correct label. Distractor options were carefully crafted to be plausible yet incorrect, following established principles for multiple-choice question design in domain-specific contexts. For each correct answer, three distractors were generated using the following strategies: (1) Semantic similarity: options that share visual or conceptual features with the correct answer but differ in key domain-specific details; (2) Common misconceptions: answers that reflect frequent errors or oversimplifications made by non-experts when interpreting opera elements; and (3) Category confusion: options from the same broad category but representing different subcategories or regional variants. For example, in a facial makeup symbolism question showing a character with predominantly red face paint, distractors might include “loyalty and righteousness” (correct), “bravery and passion” (semantic similarity - related positive traits), “cunning and deception” (common misconception - opposite emotional valence), and “scholarly wisdom” (category confusion - different character archetype entirely). Similarly, for costume attribution questions, distractors would include costumes from the same historical period but different social ranks, or visually similar elements from different opera traditions.

To further assess question-design quality beyond answer-label correctness, we conducted an additional expert review focusing on question clarity, distractor plausibility, and cultural appropriateness. A randomly selected subset of 10% of the dataset was independently evaluated by three domain reviewers using a five-point Likert scale for each dimension. Question clarity assessed whether the question was linguistically clear and answerable based on the image and relevant cultural knowledge. Distractor plausibility assessed whether the incorrect options were reasonable and non-trivial while remaining clearly distinguishable from the correct answer. Cultural appropriateness assessed whether the question and answer choices accurately reflected traditional Chinese opera conventions without introducing misleading or oversimplified interpretations. Items with low ratings or inconsistent reviewer judgments were further discussed and revised through expert consensus before final inclusion.

To support bilingual access and cross-lingual use, all benchmark questions were made available in both Chinese and English. In terms of original question construction, 624 items were first authored in English or contain English original question text, while 376 items were first authored in Chinese or other non-ASCII text and include corresponding English translation fields. This distribution describes only the original authored language of each item and should not be interpreted as defining two independently balanced language-specific evaluation sets. For cross-lingual analysis, we therefore used the 376 Chinese-original items that contain paired English versions. In this paired subset, each Chinese input and its English counterpart share the same image, answer label, option structure, and intended cultural meaning. To reduce translation-induced bias, the bilingual versions were reviewed by annotators with both linguistic and traditional Chinese opera expertise. The review focused on semantic equivalence, preservation of culturally specific terminology, consistency of answer choices, and avoidance of literal translations that might distort opera-specific meanings. Items with potentially non-equivalent wording were revised through discussion between bilingual annotators and domain experts before final inclusion.

Every annotated sample underwent a three-step validation process: expert review for factual and cultural correctness, linguistic review for clarity and balance of distractors, and a final formatting check for compliance with encoding and structural requirements. This combination of expert curation and structured quality control ensured that each instance met the standards for evaluating multimodal reasoning in a culturally complex setting.

Because each sample required image inspection, question construction, distractor design, bilingual review, and final validation, the annotation process was conducted manually on an item-by-item basis rather than through automatic template generation. This workflow ensured high cultural fidelity and interpretive quality, although it also made dataset construction labor-intensive.

### Data Organization

**Fig. 2:**
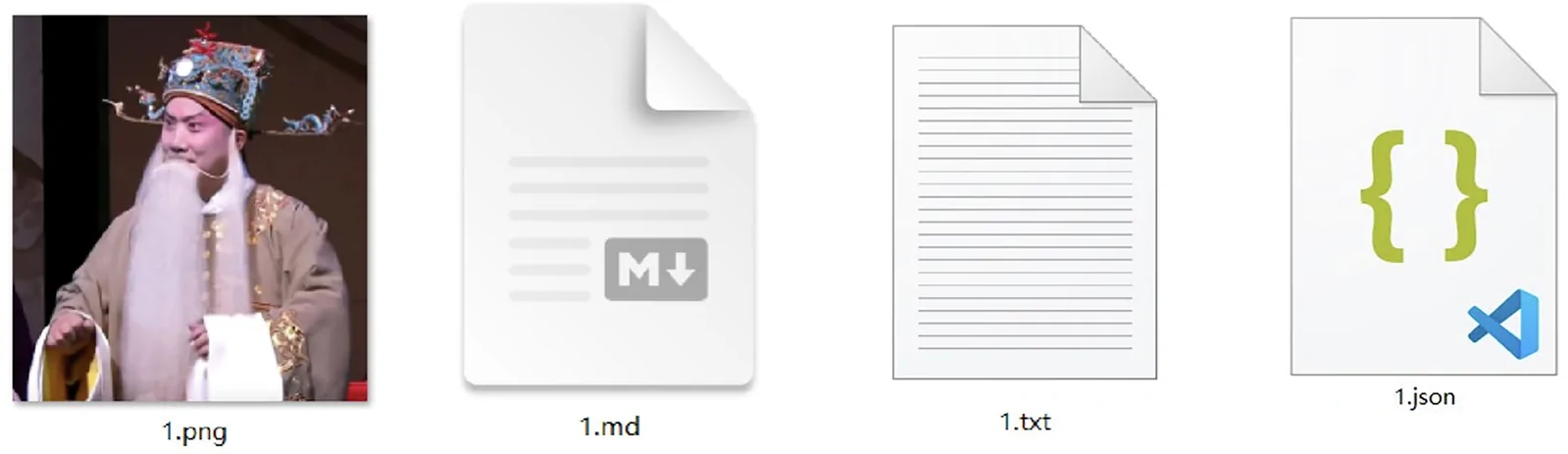
TCO-Dataset file format.

The TCO-Dataset is organized to ensure ease of use for both human evaluators and machine learning systems. Each data sample comprises four files: a high-resolution image in PNG format, a Markdown file containing the multiple-choice question, a plain text file indicating the correct answer label, and a structured JSON file consolidating all metadata for the sample. All files are named using a consistent numeric identifier (e.g., 0001.png, 0001.md, 0001.txt, 0001.json) and stored in a single-level directory structure for straightforward indexing and batch processing, as illustrated in Fig. 2.

All images are in PNG format to preserve quality and ensure lossless compression, which is critical for capturing fine-grained visual details such as facial makeup patterns and costume textures. The corresponding Markdown files contain a single question followed by four answer choices labeled (A) through (D). The question format is consistent across samples to support automated parsing. The correct answer is specified in the accompanying ‘.txt’ file using a single uppercase letter (e.g., D), which facilitates direct integration into evaluation pipelines without the need for additional preprocessing.

The ‘.json’ files encapsulate all relevant information for each sample in a structured format, including the question text, answer choices, ground-truth label, and file references. This enables convenient programmatic access and is especially useful for automated benchmarking, dataset visualization, or API-based model evaluation.

The 1,000 samples are evenly distributed across the eight opera genres featured in the dataset: Henan Opera (100), Huangmei Opera (200), Qinqiang (100), Peking Opera (200), Cantonese Opera (100), Yue Opera (100), Sichuan Opera (100), and Kunqu Opera (100). Question categories are balanced across these genres to ensure diversity in visual and cultural reasoning tasks.

### Quality Assurance and Validation

To ensure the cultural fidelity, linguistic clarity, and structural consistency of the TCO-Dataset, we adopted a dual-layered quality assurance framework that combines comprehensive manual expert review with automated validation procedures. This process was designed to guarantee both the semantic soundness of the annotations and the technical integrity of the dataset.

All annotated samples—including the image, question, answer choices, and correct label—were subjected to a structured three-step manual review. First, domain experts in traditional Chinese opera independently verified each question for factual accuracy, ensuring correct associations between visual elements and cultural knowledge. They examined whether features such as facial makeup, costumes, and gestures were appropriately linked to specific character roles or historical contexts. Second, bilingual reviewers evaluated the language quality of both Chinese and English questions, checking for fluency, clarity, and semantic precision. Special care was taken to design distractor options that were plausible yet clearly distinct from the correct answer. Third, each sample underwent a formatting and consistency check, confirming adherence to the dataset’s structural schema, including proper Markdown syntax, standardized answer labeling, and consistent file naming.

To further validate annotation consistency, we randomly selected 10% of the dataset for blind re-annotation by independent experts not involved in the initial process. This cross-review yielded a Cohen’s Kappa score of 0.87, indicating a high level of inter-annotator agreement and confirming the reliability of the annotation protocol. In addition to answer-label agreement, the same subset was further assessed for question-design quality. The average expert ratings were 4.58 ± 0.52 for question clarity, 4.36 ± 0.59 for distractor plausibility, and 4.55 ± 0.51 for cultural appropriateness. Inter-rater consistency was measured using the intraclass correlation coefficient (ICC), yielding values of 0.88, 0.88, and 0.89 for the three dimensions, respectively. These results indicate that the questions were generally clear, the distractors were plausible without being ambiguous, and the cultural interpretations were stable across independent expert reviewers. Items with lower ratings mainly involved overly broad wording, distractors that were either too obvious or too close to the correct answer, and cultural explanations that required more precise terminology. These cases were revised through expert consensus before the final dataset release. Analysis of the disagreement cases (13% of re-annotated samples) revealed three primary sources of inconsistency: (1) Genre disambiguation ambiguity - cases where visual features could plausibly indicate multiple opera genres, particularly between closely related regional variants (e.g., distinguishing Huangmei Opera from Yue Opera when costume details were similar); (2) Character role classification uncertainty - instances where traditional role categories (Sheng, Dan, Jing, Chou) were ambiguous due to cross-gender performances or hybrid character types that blend multiple role conventions; and (3) Temporal period attribution disputes - disagreements regarding the historical era of costumes or props, especially for pieces that blend traditional and contemporary design elements.

Disagreement resolution followed a structured consensus-building process. For each disputed case, the original annotator and the independent reviewer jointly re-examined the sample alongside a third domain expert. The resolution process prioritized cultural authenticity and visual evidence hierarchy, where clear visual indicators (e.g., distinctive makeup patterns, region-specific costume elements) took precedence over subjective interpretations. Most disagreement cases were resolved through expert discussion and revision of the annotation record, while a very small number of items for which stable expert consensus could not be reached (2 samples) were excluded from the final dataset. This process ensured that the released benchmark retained only items that met the required standard of interpretive reliability.

In parallel, a series of automated scripts were applied to identify and correct structural anomalies. These included checks for missing or unmatched files, invalid or improperly formatted answer labels, encoding inconsistencies, and incomplete question structures. All flagged items were manually inspected and resolved prior to dataset release.

### Ethics Statement

The data collection involving human subjects (field photography) was conducted with strict adherence to ethical standards. All performers granted written informed consent for their likenesses to be used for research purposes and for inclusion in the released benchmark dataset. The study protocol regarding data collection was reviewed and approved by the Academic Committee of the School of Automation Science and Engineering, Xi’an Jiaotong University. The released benchmark was prepared from source materials curated by the authors and their affiliated institution under institutional oversight.

## Data Records

TCO-Dataset is available on Hugging Face (https://doi.org/10.57967/hf/7744)^22^. The dataset repository ensures long-term persistence and public access under the CC-BY 4.0 license. This license permits use, sharing, adaptation, distribution, and reproduction in any medium or format, including commercial use, provided the original work is properly cited.

External users may access the released benchmark dataset through the public Hugging Face dataset page (https://huggingface.co/datasets/WilliamHS/TCO-Dataset) and its DOI-linked repository record (https://doi.org/10.57967/hf/7744). The broader internal archival materials from which the benchmark was curated are institutionally managed and are not presented in this work as a separate public third-party repository.

**Fig. 3:**
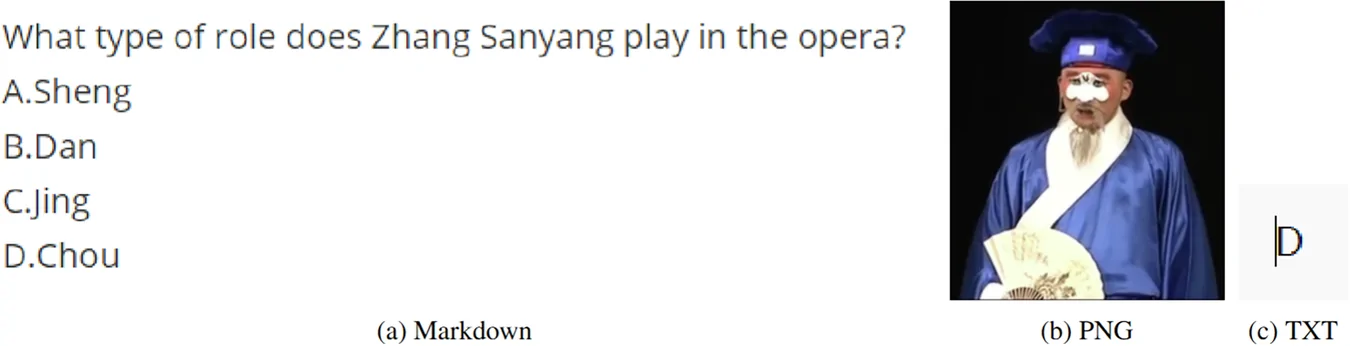
Example TCO-Dataset item.

This structured format ensures compatibility with standard multimodal evaluation pipelines, as shown in Figs. 2, 3. To support future domain adaptation and fine-tuning research, we additionally provide standardized dataset splits. The 1,000 samples are divided into training, validation, and test subsets containing 700, 100, and 200 samples, respectively. The split was generated using a fixed random seed over the released sample identifiers to provide a reproducible and consistent data protocol for future studies. The split files are provided as train.json, val.json, and test.json, with each file listing the sample identifiers assigned to the corresponding subset. A split_metadata.json file documents the split size, generation method, random seed, and intended usage. Because the released JSON metadata do not currently contain per-sample labels for opera genre, question type, difficulty level, or original authored language, this split is intended as a reproducible standard split rather than a strictly metadata-stratified split. These standardized splits are intended to facilitate supervised fine-tuning, validation during domain adaptation, and fair comparison of future fine-tuned models on a held-out test set.

The JSON metadata files serve as the primary entry point for programmatic access and rigorous data traceability. Within each.

JSON object, the id variable provides the unique identifier that links all associated files. The filenames for the visual and textual components are explicitly referenced in the image, question_file, and answer_file fields, respectively. The semantic content of the query is stored in the question_text field, while the candidate answers are organized in a nested options dictionary (keyed by A through D). To support bilingual access, the metadata provide translation fields that allow each benchmark item to be viewed in both Chinese and English. These fields are auxiliary metadata used to improve accessibility and cross-lingual usability, and should be distinguished from the original language in which the question was first authored. The ground truth label is designated by the answer field. To ensure rigorous data provenance without introducing file-level redundancy, the source and licensing information are explicitly declared at the repository level. The dataset’s README file serves as the master metadata document, confirming that all samples in the released benchmark were curated from primary materials collected, digitized, or documented by the authors and their affiliated institution and released under the CC-BY 4.0 license. This ensures that provenance is globally traceable for all 1,000 samples.

The dataset covers eight major opera genres that are representative of the regional, linguistic, and stylistic diversity of Chinese opera. Genre-specific sample counts are summarized in Table 1. Across the released dataset, 624 items contain English-original or ASCII-authored question text, while 376 items contain Chinese or other non-ASCII original question text and paired English translation fields.

**Table 1:** TCO-Dataset statistics.

Opera Type	Total Samples
Henan Opera	100
Huangmei Opera	200
Qinqiang Opera	100
Peking Opera	200
Cantonese Opera	100
Yue Opera	100
Sichuan Opera	100
Kunqu Opera	100
**Total**	1000

**Fig. 4:**
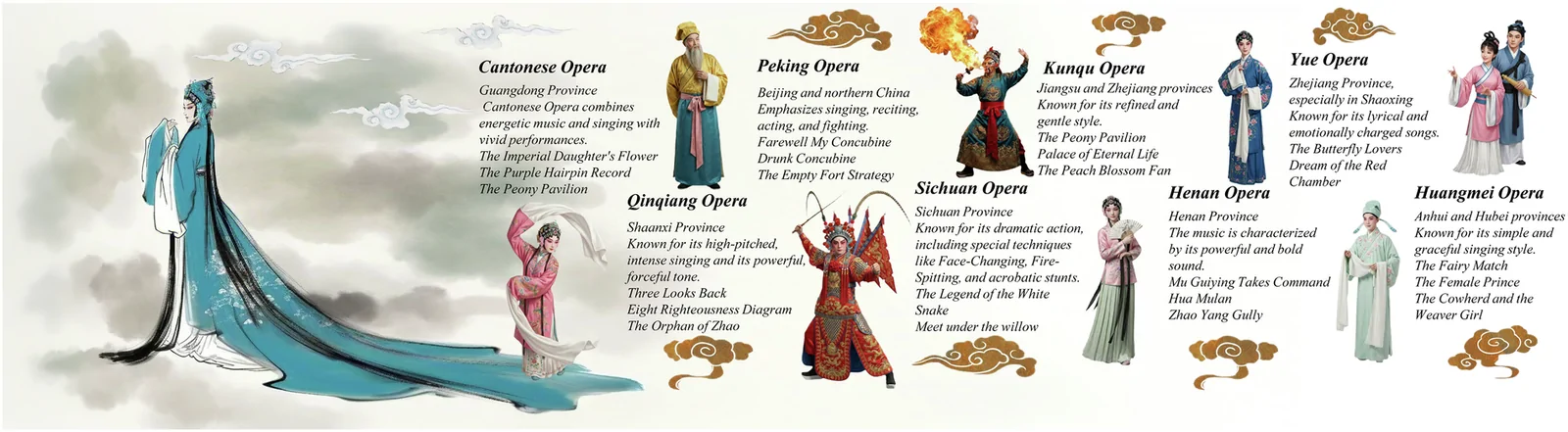
Evaluation task overview.

Each sample includes a question targeting one of several core aspects of opera understanding, including genre classification, costume period, instrument recognition, character roles (e.g., Sheng, Dan, Jing, Chou), emotional tone, and opera story identification. All benchmark questions are available with Chinese and English fields in the metadata. The distribution of 624 English-original items and 376 Chinese/non-ASCII-original items refers only to the original authored language of the questions, rather than to two fully equivalent and independently balanced evaluation sets. The auxiliary bilingual fields are intended to improve accessibility and to support future paired cross-lingual analysis. However, language-specific performance comparisons should be interpreted with caution unless they are conducted on strictly matched bilingual subsets with identical image, answer label, option structure, and intended cultural meaning. The representative opera genres covered by the dataset are shown in Fig. 4.

## Technical Validation

### Human Expert Validation

The dataset underwent three rounds of expert review, with each sample independently checked by specialists in traditional Chinese opera, including university professors and professional performers. The review process involved consistency checks across question categories and image content, and ambiguous cases were revised or removed based on expert feedback to ensure cultural and factual integrity.

Taken together, these validation procedures support the use of TCO-Dataset as a culturally grounded benchmark for evaluating multimodal reasoning on traditional Chinese opera imagery.

### Baseline Evaluation Setup and Initial Findings

To validate the dataset’s challenge level and utility, we conducted large-scale evaluations on nine state-of-the-art MLLMs, including GPT-4o^23^, GPT-4o-mini^23^, Qwen2-VL-72B/7B^8^, LLaVA:7B^24^, LLaMA3.2-vision:11B^24^, InternVL2-26B^9^, and others. Given an opera still and an associated question, each model was tasked with selecting the correct answer from four choices. We use accuracy as the primary evaluation metric. To additionally reflect output-format robustness under constrained answer generation, we also report the valid response rate, defined as the proportion of model outputs from which a valid answer choice (A, B, C, or D) could be reliably extracted. Results indicate substantial room for improvement. The top-performing model, Qwen2-VL-72B-Instruct, achieved an F1 score of 0.73, while GPT-4o and LLaVA:7B trailed at 0.59 and 0.44 respectively. The average model performance across all systems was considerably lower than on standard VQA datasets, highlighting the challenges posed by cultural reasoning tasks. Our error analysis indicates that model failures are concentrated in several recurring visual-cultural reasoning scenarios, including genre disambiguation among visually similar opera traditions, interpretation of symbolic facial makeup, role-category recognition, historical costume attribution, and contextual grounding in regional or narrative conventions. These patterns suggest that model errors are not merely caused by generic image-recognition difficulty, but are closely related to the need to connect fine-grained visual cues with culturally specific knowledge.

### Evaluation Objectives

The primary objective of this evaluation is to validate the effectiveness, difficulty, and utility of the TCO-Dataset as a benchmark for assessing the multimodal reasoning capabilities of large language models (LLMs), particularly in culturally rich domains such as traditional Chinese opera. Unlike general visual question answering datasets that focus on everyday objects or scientific diagrams, TCO-Dataset introduces a set of tasks that require both fine-grained visual understanding and deep cultural-historical knowledge.

The evaluation framework is structured around two interdependent dimensions: Image Comprehension and Knowledge Reasoning. To operationalize these dimensions more explicitly, we mapped each question type to a primary evaluation dimension according to the dominant capability required for answering. Image Comprehension refers to questions in which the main challenge lies in extracting and interpreting visually observable elements from the still image, such as opera genre, costume features, visible props, instruments, role appearance, and facial makeup patterns. Knowledge Reasoning refers to questions in which the main challenge lies in connecting visual cues with domain-specific cultural, historical, regional, or narrative knowledge, such as symbolic interpretation of facial makeup, plot/story identification, historical costume attribution, emotional tone inference, and regional tradition awareness.

**Fig. 5:**
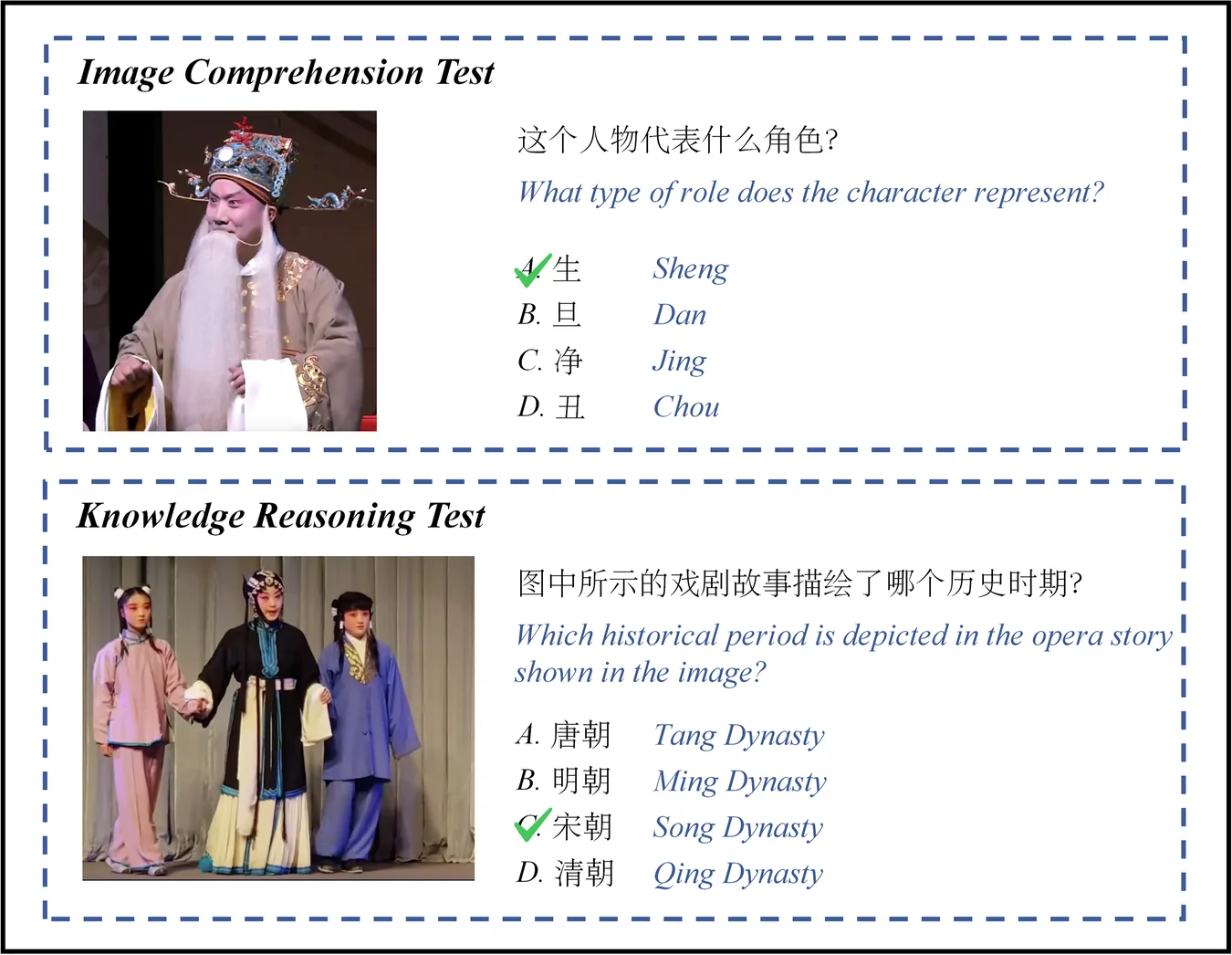
Image comprehension and knowledge reasoning examples.

Because traditional Chinese opera understanding often requires both visual perception and cultural knowledge, these two dimensions should not be regarded as strictly orthogonal. Instead, each item was assigned a primary dimension for analytical clarity, while acknowledging that many questions involve both visual evidence and background knowledge. For example, role appearance identification is primarily categorized as Image Comprehension when the answer can be inferred from visible costume and makeup cues, whereas facial makeup symbolism is categorized as Knowledge Reasoning when the task requires interpreting the cultural meaning of color and pattern conventions. This operationalization allows the benchmark to report dimension-level tendencies without assuming complete independence between the two dimensions. Examples of these two dimensions are shown in Fig. 5.

Together, these two components present a focused still-image reasoning challenge to LLMs, requiring not only accurate image-text alignment but also the ability to reason through multi-step inferences embedded within a historical and symbolic framework. By design, the questions in TCO-Dataset span diverse opera genres, question types, difficulty levels, character genders, and linguistic contexts (English and Chinese), allowing for multi-faceted evaluation of cross-modal generalization, cultural adaptation, language sensitivity, and reasoning proficiency across skill levels.

### Evaluation Metrics

We evaluate model performance primarily using accuracy, which is the proportion of questions for which the extracted model prediction matches the ground-truth answer label. Because TCO-Dataset is formulated as a single-choice multiple-choice benchmark with exactly one correct option per question, accuracy provides the most direct and interpretable measure of task performance.

To additionally characterize response-format robustness, we report the valid response rate. This metric is defined as the proportion of model outputs from which a valid option label (A, B, C, or D) can be successfully extracted under the predefined parsing rules. Outputs that do not yield a valid option are treated as invalid responses.

Formally, let N denote the total number of questions, let ŷ_*i*_ denote the extracted prediction for question_*i*_, and let y_*i*_ denote the corresponding ground-truth answer. Then accuracy is computed as the fraction of questions for which ŷ_*i*_ = y_*i*_. Let V denote the number of outputs from which a valid option label can be extracted. The valid response rate is computed as V / N.

This metric design separates two aspects of performance: task correctness, captured by accuracy, and output-format compliance, captured by valid response rate. We adopted this formulation to avoid ambiguity in interpreting precision, recall, and F1 score in a single-choice benchmark setting.

For answer extraction, the evaluation script normalized each model output by removing a trailing period and converting the response to uppercase. The output was accepted as a valid prediction only if it exactly matched one of the four option labels, A, B, C, or D. Responses containing explanations, multiple option letters, or any text other than a single valid option label were marked as invalid. Invalid responses were counted as incorrect in the accuracy calculation and were also reflected in the valid response rate.

Statistical Analysis. To provide a more robust basis for cross-model comparison, we supplemented the point estimates of accuracy with confidence intervals, paired significance testing, and category-specific analysis. For each model, we computed 95% confidence intervals for accuracy using non-parametric bootstrap resampling over benchmark items. Specifically, we resampled the 1,000 benchmark items with replacement for 10,000 iterations and recalculated accuracy for each resampled set. The 2.5th and 97.5th percentiles of the bootstrap accuracy distribution were reported as the lower and upper bounds of the 95% confidence interval.

To assess whether observed differences between models were statistically significant, we used paired McNemar’s tests based on item-level correctness. This test is appropriate because all models were evaluated on the same set of questions, allowing direct comparison of paired correct/incorrect outcomes. For multiple pairwise comparisons, p-values were adjusted using the Holm-Bonferroni correction. We report adjusted p-values for the main comparisons between the top-performing model and representative API-based and open-source baselines.

In addition, we conducted category-specific analysis by opera genre and question type. For each subgroup, we computed model accuracy and examined whether performance differences were consistent across the dataset or concentrated in specific cultural or visual categories. This analysis helps identify which opera genres and reasoning types pose the greatest challenges for current multimodal models.

### Baseline Models

To establish baseline performance on the TCO-Dataset, we selected nine representative multimodal large language models (MLLMs) spanning a range of architectures, parameter sizes, and training paradigms. These models include both proprietary (API-based) and open-source systems, allowing for comprehensive evaluation across development ecosystems and deployment settings.

The proprietary models in our evaluation include Qwen2-VL-72B-Instruct^8^, GPT-4o^23^, and GPT-4o-mini^23^. Qwen2-VL-72B-Instruct is a 72-billion-parameter model developed by Alibaba, instruction-tuned for high-resolution image and language integration tasks. GPT-4o is OpenAI’s flagship multimodal model with strong cross-modal reasoning capabilities. Its lightweight counterpart, GPT-4o-mini, offers lower computational overhead while maintaining competitive reasoning performance.

The open-source models evaluated include Qwen2-VL-7B-Instruct^8^, LLaVA:7B^24^, LLaVA-LLaMA3:8B^24^, LLaVA-Phi3:3.8B^24^, LLaMA3.2-Vision:11B^24^, and InternVL2-26B^9^. These models represent a broad spectrum of recent community-driven MLLM development. For example, LLaVA:7B is built on the LLaMA2 backbone with visual instruction tuning, while LLaVA-LLaMA3:8B and LLaVA-Phi3:3.8B explore newer architectures combining visual grounding with compact or general-purpose language models. InternVL2-26B, a model developed in China, emphasizes structured multimodal input and domain adaptation. These models vary in parameter size from 3.8B to 26B and in training corpus composition, providing a robust test bed for cross-architecture and cross-domain comparison^25,26^.

All selected models support both visual and textual inputs and were run in a zero-shot setting without fine-tuning. This diversity enables a realistic comparison of model generalization, robustness, and their ability to handle culturally complex multimodal tasks such as those presented in TCO-Dataset.

### Evaluation Setup

All models were evaluated under a consistent zero-shot setting, without any fine-tuning, prompt tuning, or in-context learning on the TCO-Dataset. This zero-shot setting was used as the baseline evaluation protocol in the present study. In addition to this baseline protocol, the released dataset now includes standardized training, validation, and test splits to support future supervised fine-tuning and domain adaptation experiments. We did not fine-tune the evaluated models in this work, in order to preserve a clean assessment of their out-of-the-box visual-cultural reasoning capabilities. Therefore, the zero-shot results reported in this paper were computed on the full 1,000-item benchmark rather than only on the test split. Each model was required to process the entire set of 1,000 image-question pairs and return a single-choice answer from four options (A, B, C, D).

For each sample, the evaluation script loaded three files with the same numeric identifier: the Markdown question file, the plain-text answer file, and the PNG image file. The first line of the Markdown file was parsed as the question, and the following four lines were parsed as options A, B, C, and D. The corresponding TXT file provided the ground-truth answer label. This file-based loading strategy ensured that all models were evaluated using the same image-question-answer records.

For proprietary models (GPT-4o, GPT-4o-mini, and Qwen2-VL-72B-Instruct), we accessed the official inference APIs provided by OpenAI and Alibaba. For open-source models, we employed their recommended local inference frameworks, including Hugging Face Transformers for LLaVA variants and Qwen2, and the InternVL inference interface for InternVL2-26B. All local experiments were conducted on a high-performance server equipped with an NVIDIA A100 GPU (80GiB VRAM), 256GB RAM, and PyTorch version 2.1.For image input, the evaluation script loaded the original PNG file of each sample and encoded it directly into a base64 data URI for model inference. We did not apply additional manual resizing, cropping, tiling, or aspect-ratio distortion in the evaluation script. Any model-specific resizing, compression, normalization, or patch construction was handled internally by the corresponding API endpoint or by the official image processor used by the local inference framework. This procedure preserves the released image content while avoiding an additional hand-crafted preprocessing stage that could favor a particular model family.

All generations were run deterministically. In the evaluation script, the temperature was set to 0 and the maximum output length was limited to 10 tokens, because the task required only a single option label. No retries, self-consistency sampling, or manual correction was applied during evaluation.

Each input consisted of a high-resolution opera still image and a corresponding multiple-choice question. To unify output formatting across different model architectures, all models were instructed to return only the selected option letter from A, B, C, or D, without any explanation or additional text. The complete prompt template used in the evaluation script was as follows:

“根据下面这张图片回答这个选择题:

{question_text}

A: {option_A}

B: {option_B}

C: {option_C}

D: {option_D}

请仅回答正确选项的字母(A、B、C 或 D), 不允许有其他任何内容。”

**Fig. 6:**
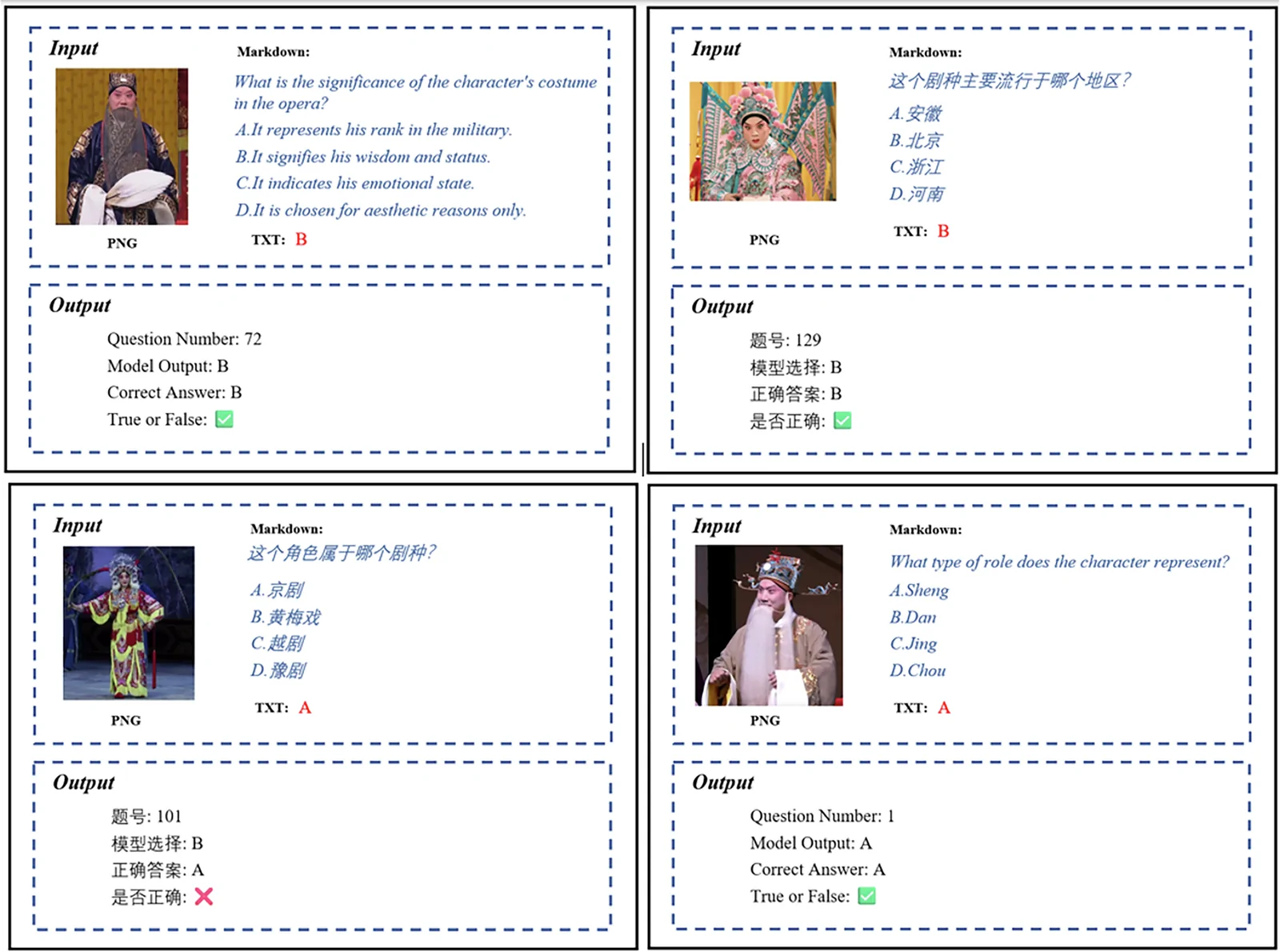
Model input and answer format.

For English-language evaluation, the same template structure was used with the English question and option texts. The answer-format instruction was kept equivalent across languages: the model was required to output only a single option label, A, B, C, or D. After inference, the evaluation script generated separate structured records for scoring and reporting, including the extracted model prediction, the ground-truth answer, and the correctness result. These post-processing records were not part of the JSON output requested from the model. The model input and constrained answer format are illustrated in Fig. 6.

This format facilitates automatic answer extraction and ensures consistency in evaluation. For post-processing, we used a JSON-first and context-anchored answer extraction procedure. The evaluation script first attempted to parse the model output as a JSON object and extract the value associated with the prediction field. If the parsed value was exactly one of A, B, C, or D, it was accepted as the model prediction. If JSON parsing failed, the script then applied a conservative fallback parser that accepted only explicitly anchored answer expressions, including “prediction: A/B/C/D”, “answer: A/B/C/D”, “final answer: A/B/C/D”, or a standalone option label appearing alone on a line. Importantly, the parser did not match arbitrary uppercase letters inside words or explanatory sentences. For example, letters appearing in words such as “Correct” or in phrases such as “Choice A is wrong” were not treated as model predictions. Outputs that did not yield a valid option under these rules were marked as invalid and counted as incorrect in the accuracy calculation, while also being reported through the valid response rate.

To ensure fairness, all models were evaluated using the same scoring script and the same metric definitions—accuracy as the primary task-performance metric and valid response rate as the auxiliary output-format metric—on the full dataset.

Human Baseline Protocol. To contextualize benchmark difficulty and estimate the remaining headroom between current MLLMs and human performance, we conducted a small-scale human baseline evaluation. We randomly sampled 100 items from the TCO-Dataset while preserving approximate coverage of opera genres, question types, difficulty levels, and original authored languages. The human baseline involved two groups of participants: three domain experts with formal training or professional experience in traditional Chinese opera, and ten non-expert participants with general university-level education but no specialized training in Chinese opera. Each participant answered the sampled multiple-choice questions independently using the same image and answer options as those provided to the models. No participant had access to the ground-truth answer labels during evaluation. The same sampled item set and answer format were used for all human participants, allowing the human baseline to contextualize task difficulty and model headroom.

### Main Results

Table 2 presents the main results on TCO-Dataset, including accuracy, 95% confidence intervals, and valid response rate. Overall, the performance of the nine models varied substantially, with the best-performing model, Qwen2-VL-72B-Instruct, achieving an accuracy of 0.73 with a 95% confidence interval of [0.70, 0.76]. Most models scored between 0.11 and 0.59, indicating that current large multimodal models still face substantial challenges in understanding and reasoning about traditional Chinese opera imagery, especially with respect to complex cultural context, historical knowledge, and emotional nuance. The confidence intervals provide an estimate of the uncertainty associated with each model’s performance under the 1,000-item benchmark. However, most of the models scored between 0.11 and 0.59, which is still relatively low, suggesting that large language models (LLMs) face considerable challenges in understanding and reasoning about Chinese opera, especially with respect to the complex cultural context, historical knowledge, and emotional nuances inherent in these works.

**Table 2:** Main results on TCO-Dataset, reported using accuracy, 95% confidence intervals, and valid response rate. Part 1: API-based models; Part 2: open-source models.

Model types	Models	Accuracy	95% CI	Valid Response Rate
API-based models	Qwen2-VL-72B-Instruct	0.73	[0.70, 0.76]	0.99
	GPT-4o	0.59	[0.56, 0.62]	1
	GPT-4o-mini	0.53	[0.50, 0.56]	0.99
Open-source models	Qwen2-VL-7B-Instruct	0.56	[0.53, 0.59]	0.98
	LLaVA:7b	0.44	[0.41, 0.47]	0.95
	LLaMA3.2-vision:11b	0.36	[0.33, 0.39]	0.94
	InternVL2-26B	0.25	[0.22, 0.28]	0.88
	LLaVA-LLaMA3:8b	0.19	[0.17, 0.22]	0.83
	LLaVA-phi3:3.8b	0.11	[0.09, 0.13]	0.79

Specifically, the best-performing model, Qwen2-VL-72B-Instruct, achieved an accuracy of 0.73, demonstrating stronger performance in handling Chinese opera images and the associated cultural knowledge than the other evaluated models. In contrast, GPT-4o achieved an accuracy of 0.59, while GPT-4o-mini achieved an accuracy of 0.53. Open-source models showed more limited capabilities, with accuracy values ranging from 0.11 to 0.56. These results suggest that current MLLMs vary substantially in their ability to interpret culturally specific visual content, even under the same evaluation protocol.

The paired significance tests further support the observed performance differences. Using McNemar’s test with Holm-Bonferroni correction, the performance advantage of Qwen2-VL-72B-Instruct over GPT-4o was statistically significant (adjusted p < 0.001). Its advantage over GPT-4o-mini and the evaluated open-source baselines was also statistically significant (adjusted p < 0.001 for the main comparisons). These results suggest that the observed ranking differences are not solely due to sampling fluctuation within the 1,000-question benchmark.

Human Baseline Results. The human baseline provides additional context for interpreting model performance. On the 100-item sampled subset, domain experts achieved an average accuracy of 0.91, while non-expert participants achieved an average accuracy of 0.54. The best-performing model, Qwen2-VL-72B-Instruct, achieved an accuracy of 0.73 on the full benchmark and showed comparable but still lower performance than domain experts on the human-baseline subset. This result suggests that TCO-Dataset is answerable by knowledgeable human evaluators while still leaving substantial headroom for current MLLMs, particularly in culturally specialized reasoning Table 3.

**Table 3:** Human baseline accuracy on the sampled evaluation subset.

Evaluator group	Number of participants	Evaluation subset	Accuracy
Domain experts	3	100 sampled items	0.91
Non-expert participants	10	100 sampled items	0.54
Qwen2-VL-72B-Instruct	—	Same sampled items	0.73
GPT-4o	—	Same sampled items	0.59

The lowest-performing models, such as LLaVA-phi3:3.8b (accuracy = 0.11) and LLaVA-LLaMA3:8b (accuracy = 0.19), highlighted the substantial limitations in understanding and generating correct answers based on Chinese opera images. The performance across the board indicates significant challenges in multimodal reasoning, especially within the domain of traditional Chinese opera.

### Category-specific Performance Analysis and Error Interpretation

To further examine whether model performance was consistent across different types of opera content, we conducted category-specific analysis by opera genre and question type. Across opera genres, models generally performed better on visually distinctive and well-documented genres such as Peking Opera and Huangmei Opera, while lower accuracy was observed for genres with more subtle regional visual differences, such as Yue Opera and Kunqu Opera. This pattern suggests that model errors are not uniformly distributed across the benchmark, but are partly associated with the visual distinctiveness, cultural familiarity, and regional specificity of particular opera traditions.

A closer inspection of incorrect responses revealed several recurring sources of error. First, genre-confusion errors often occurred when models relied on visually salient but non-diagnostic cues, such as bright costume colors or generalized stage makeup, rather than region-specific costume structure, performance context, or character styling. This explains why visually adjacent opera traditions may be confused even when the image quality is high. Second, facial-makeup-related errors were typically associated with overgeneralized interpretations of color symbolism. For example, models sometimes recognized that facial makeup carried symbolic meaning, but failed to distinguish between role identity, moral symbolism, and emotional tone. Third, costume and role-recognition errors often reflected a mismatch between everyday visual assumptions and the stylized conventions of traditional Chinese opera, especially in cases involving cross-gender performance, hybrid role presentation, or historically specific costume elements.

Question-type analysis further showed that models performed relatively better on direct visual recognition tasks, such as prop recognition and role appearance identification, but struggled more with questions requiring symbolic or cultural reasoning, such as facial makeup interpretation, plot/story identification, and historical costume attribution. These findings indicate that TCO-Dataset not only measures overall multimodal accuracy, but also reveals more fine-grained weaknesses in models’ ability to connect visual evidence with domain-specific cultural knowledge. The category-specific results are summarized in Table 4. The dimension-level mapping in Table 5 further clarifies whether each question type primarily evaluates image comprehension or knowledge reasoning, while recognizing that these dimensions are analytically useful but not fully independent.

**Table 4:** Category-specific accuracy analysis by opera genre.

Opera genre	Qwen2-VL-72B-Instruct	GPT-4o	GPT-4o-mini	Qwen2-VL-7B-Instruct	LLaVA:7B
Henan Opera	0.72	0.58	0.52	0.55	0.43
Huangmei Opera	0.76	0.61	0.55	0.59	0.46
Qinqiang Opera	0.71	0.57	0.51	0.54	0.42
Peking Opera	0.79	0.64	0.58	0.62	0.49
Cantonese Opera	0.72	0.58	0.52	0.55	0.43
Yue Opera	0.68	0.55	0.49	0.52	0.4
Sichuan Opera	0.74	0.6	0.54	0.57	0.45
Kunqu Opera	0.67	0.53	0.48	0.5	0.38

**Table 5:** Mapping between question types and primary evaluation dimensions.

Primary dimension	Question types	Main capability assessed	Approximate proportion
Image Comprehension	Opera genre classification; prop and instrument recognition; character role appearance identification	Recognition and interpretation of visually observable elements in opera still images	46%
Knowledge Reasoning	Historical costume attribution; facial makeup symbolism; plot/story identification; emotional tone inference; regional distribution awareness	Linking visual cues with cultural, historical, symbolic, narrative, or regional knowledge	54%

### Performance Differences between API Models and Open-source Models

In the evaluation on the TCO-Dataset, API-based closed-source models, such as Qwen2-VL-72B-Instruct, GPT-4o, and GPT-4o-mini, outperform open-source models across the board. The best-performing model, Qwen2-VL-72B-Instruct, achieved an accuracy of 0.73, significantly surpassing the performance of open-source models. In comparison, GPT-4o and GPT-4o-mini achieved accuracies of 0.59 and 0.53, respectively.

On the other hand, open-source models such as LLaMA3.2-vision:11b, LLaVA:7B, and LLaVA-LLaMA3:8B show relatively weaker performance, with accuracies of 0.36, 0.44, and 0.19, respectively. Even more pronounced is the underperformance of models like LLaVA-Phi3:3.8B and InternVL2-26B, which recorded much lower accuracies of 0.11 and 0.25.

The substantial performance gap between API-based and open-source models can be attributed to several interconnected factors that reflect fundamental differences in development resources and training strategies:

Training Data Scale and Quality: API-based models like Qwen2-VL-72B-Instruct and GPT-4o likely benefited from significantly larger and more diverse multimodal training corpora, including extensive Chinese cultural content. Proprietary models typically have access to curated, high-quality datasets that may include licensed cultural archives, professional photography, and expert-annotated materials—resources that are often unavailable to open-source projects due to licensing constraints.

Architectural Sophistication: The performance hierarchy (Qwen2-VL-72B > GPT-4o > smaller open-source models) suggests that API-based models employ more sophisticated vision-language fusion mechanisms optimized specifically for cross-modal reasoning. These models likely incorporate advanced attention mechanisms, multi-scale visual feature extraction, and specialized cultural knowledge integration components that require substantial computational resources to develop and validate.

Cultural-Specific Training: Chinese opera understanding demands exposure to culturally-specific visual patterns, symbolic systems, and contextual knowledge that may be underrepresented in general-purpose training datasets. API-based models, particularly Qwen2-VL developed by Alibaba, likely incorporated targeted Chinese cultural content during training, including opera-related materials, historical texts, and cultural imagery that enhance domain-specific reasoning capabilities.

### Relationship Between Model Performance and Parameter Size

In this evaluation, a positive correlation between model parameter size and performance on the TCO-Dataset was observed. Larger models generally performed better: for example, Qwen2-VL-72B-Instruct (72B parameters) achieved an accuracy of 0.73, while GPT-4o scored 0.59, and GPT-4o-mini recorded 0.53. In contrast, smaller models such as LLaVA-phi3:3.8b (3.8B parameters) and LLaVA-LLaMA3:8b (8B parameters) achieved accuracies of 0.11 and 0.19, respectively.

This pattern suggests that larger models, with more parameters, tend to exhibit stronger performance due to their greater computational capacity and ability to capture more complex features. However, this relationship is not strictly linear. For instance, LLaVA:7b (7B parameters) outperformed some larger models, such as LLaMA3.2-vision:11b (11B parameters), achieving an F1 score of 0.44 compared to LLaMA3.2-vision’s 0.36. This indicates that other factors, such as training data quality, model architecture, and fine-tuning, also significantly influence model performance.

Although larger models generally show better results, this highlights that size alone is not a definitive factor for success in tasks like Chinese opera image understanding, where domain-specific training and optimization play a crucial role.

### Invalid Response Patterns

In this experiment, several models exhibited unstable answer-format compliance, which provides useful insight beyond overall task accuracy. In particular, some models failed to return a valid multiple-choice option in the required A/B/C/D format, even when they appeared to generate partially relevant reasoning text. Instead, these models sometimes reduced the task to binary-style responses such as “T” (True) or “N” (False), or produced other free-form outputs that could not be mapped reliably to a valid answer choice.

This behavior indicates that model failure on TCO-Dataset is not limited to deficiencies in visual-cultural reasoning alone. For some systems, an additional source of performance degradation lies in their inability to follow constrained answer-format instructions under a single-choice evaluation setting. As a result, these models show lower valid response rates and reduced overall accuracy, even when fragments of their generated text suggest partial understanding of the visual content.

**Fig. 7:**
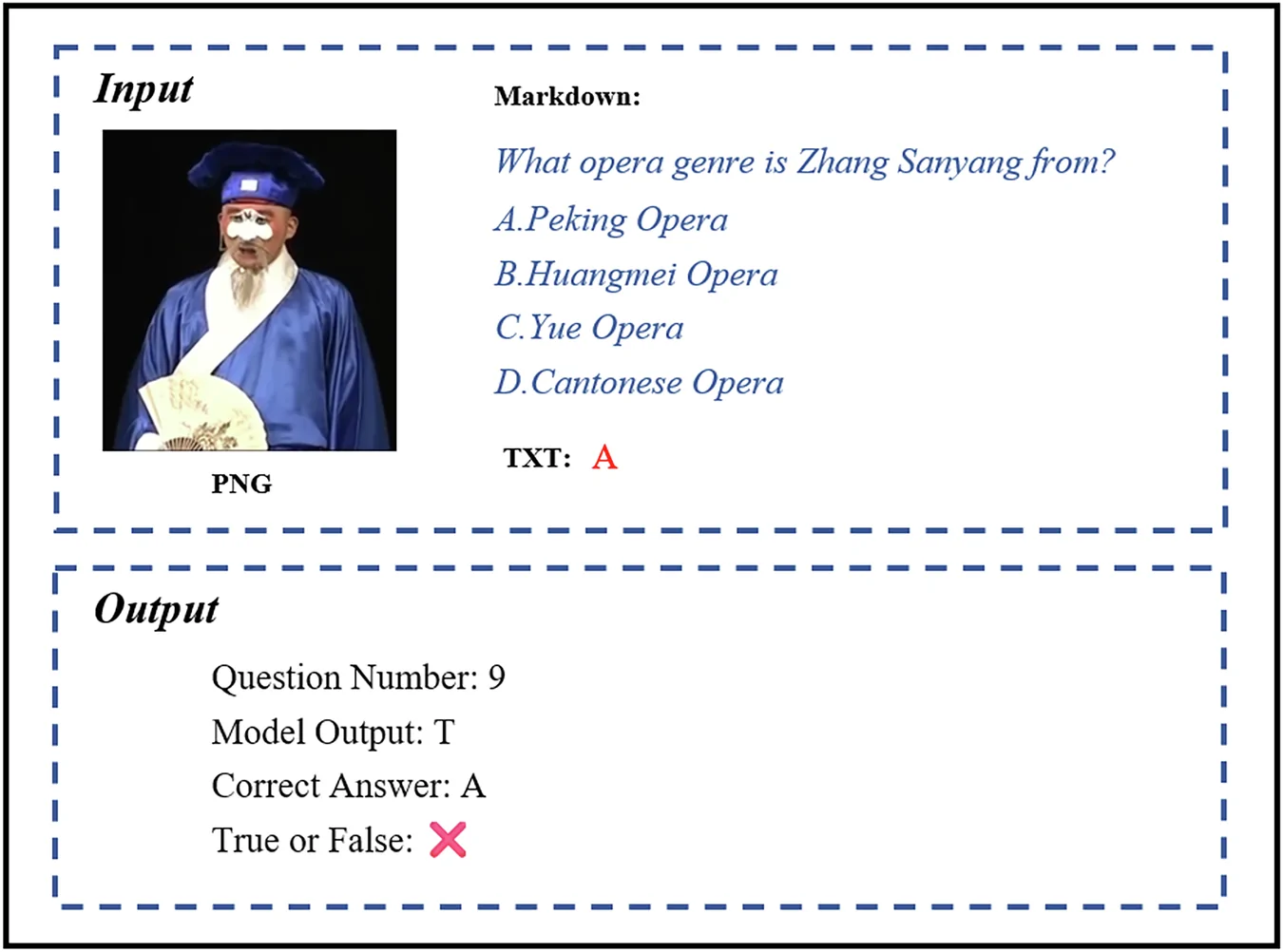
Output parsing and evaluation record.

For example, models such as InternVL2-26B, LLaVA-LLaMA3:8b, and LLaVA-phi3:3.8b were more likely to produce invalid or weakly constrained outputs than the top-performing systems. This pattern highlights a practical challenge in benchmark-based evaluation: robust performance requires not only correct reasoning over culturally grounded content, but also reliable compliance with the required response format. Figure 7 illustrates representative examples of such invalid-response patterns.

### Exploratory Analysis of Chinese and English Question Versions

To address possible content-level confounding in cross-lingual evaluation, we conducted an additional paired bilingual analysis on the 376 Chinese-original items for which paired English versions are available. Each Chinese input and its English counterpart share the same image, answer label, option structure, and intended cultural meaning. This setting allows us to compare model performance under different language presentations while controlling the evaluated item content. To avoid content-level confounding, the comparison was conducted using matched bilingual items: each Chinese input and its English counterpart shared the same image, answer label, option structure, and intended cultural meaning. Therefore, the comparison reflects differences associated with language presentation under controlled item content, rather than differences caused by unmatched question difficulty or topic distribution.In this section, we analyze the performance differences of the models when tested on Chinese and English questions from the TCO-Dataset, which are shown in Table 2. The results show notable variations, especially in how models perform on Chinese versus English questions, highlighting the influence of linguistic and cultural factors in the models’ understanding of Chinese opera.

Because each item is a single-answer multiple-choice question and each model produces one option label, recall, precision, and F1 are numerically equivalent to accuracy under this evaluation setup. The comparison is restricted to models for which both Chinese-original results and newly evaluated paired English-version results on the same 376-item subset were available.

As shown in Table 6, the paired bilingual results reveal model-dependent language effects. Qwen2-VL-72B-Instruct achieved higher performance on the Chinese-original version than on the paired English version, decreasing from 0.71 to 0.6676. By contrast, GPT-4o and GPT-4o-mini achieved slightly higher performance on the paired English versions, improving from 0.52 to 0.5771 and from 0.50 to 0.5505, respectively. These results suggest that language-related performance differences are not uniform across models. Even under paired item content, performance may reflect interactions among language presentation, translation wording, cultural terminology, and model-specific cross-lingual grounding.

**Table 6:** Paired bilingual model performance (n = 376).

Models	English			Chinese		
	Recall	Precision	F1 Score	Recall	Precision	F1 Score
Qwen2-VL-72B-Instruct	0.6676	0.6676	0.6676	0.71	0.71	0.71
GPT-4o	0.5771	0.5771	0.5771	0.52	0.52	0.52
GPT-4o-mini	0.5505	0.5505	0.5505	0.50	0.50	0.50

### Limitations and Biases Analysis

While the TCO-Dataset significantly advances culturally grounded multimodal benchmarking, we recognize the importance of articulating dataset-specific limitations, representational biases, and culture-specific challenge factors that users should consider when interpreting benchmark results. The dataset’s focus on eight major opera genres, though diverse, leads to a necessary regional and genre concentration. These prominent forms are historically more accessible and well-documented compared to the numerous local and ethnic minority operas across China. This initial focus implies that while TCO-Dataset offers a robust evaluation framework for mainstream traditional opera understanding, the generalization capabilities of models to visually distinct or lesser-known regional styles remain an important area for future investigation and dataset expansion. Furthermore, regarding gender representation and role complexity, we distinguish between numerical balance and interpretive bias. Although we sought broad statistical balance in the sampling of male- and female-associated roles, traditional Chinese opera does not always map cleanly onto mainstream visual assumptions about gender. Stylized makeup, costume conventions, and the long-standing practice of cross-gender performance may all complicate the visual presentation of role identity. As a result, models trained primarily on everyday gendered imagery may misinterpret opera-role appearance when relying on conventional gender cues. We therefore regard TCO-specific gender dynamics not only as a representational consideration in the dataset, but also as a meaningful challenge dimension for evaluating culturally grounded multimodal reasoning. This artistry can sometimes obscure simple visual cues of gender, posing a nuanced challenge for MLLMs in accurately identifying and reasoning about character roles when general training data expectations conflict with the highly specialized visual grammar of the art form.

Beyond the visual scope, we acknowledge that the current version of the TCO-Dataset relies exclusively on high-resolution still images. This design is appropriate for evaluating still-image-based visual-cultural reasoning, such as the interpretation of facial makeup, costume details, stage props, character appearance, and visually observable postures. However, it also imposes an important limitation on the scope of the benchmark. Traditional Chinese opera is fundamentally a live and time-dependent performing art that integrates movement, choreography, music, and vocal delivery. Therefore, the current dataset cannot evaluate models’ ability to understand auditory cues, musical structure, vocal technique, gesture sequences, stage movement, or the temporal development of performance. We have revised the manuscript to make this scope limitation explicit: TCO-Dataset should be understood as a benchmark for traditional Chinese opera still-image understanding rather than a complete evaluation of holistic opera comprehension. Future versions of the dataset will extend the current still-image benchmark by incorporating aligned video clips and audio materials, enabling more comprehensive evaluation of temporal, auditory, and performative dimensions of opera understanding.

## Usage Notes

For researchers interested in model adaptation, the released dataset includes standardized train/validation/test split files. The training split can be used for supervised fine-tuning or domain-adaptive instruction tuning, the validation split can be used for model selection and prompt or hyperparameter adjustment, and the test split should be reserved for final reporting. We recommend that future fine-tuning studies report results on the official test split and clearly distinguish fine-tuned performance from the zero-shot baseline results reported in this work.

The TCO-Dataset is designed as an extensible benchmark. The current version serves as a first-stage still-image benchmark that lays a foundation for evaluating visual-cultural reasoning in traditional Chinese opera. For future iterations, the most impactful extension is the incorporation of temporal and auditory dimensions. This will involve curating corresponding video and audio clips that align with the current image data, enabling researchers to evaluate MLLMs’ ability to interpret key performance elements such as vocal techniques, musical structure, gesture sequences, and choreographed movements. Furthermore, to enhance cultural coverage and address potential regional biases, future work will focus on the systematic inclusion of more regional and niche opera genres beyond the current eight. These extensions will allow the TCO-Dataset to evolve from a still-image benchmark into a multi-sensory and more geographically comprehensive resource for the field.

## Supplementary information

Supplementary information

## Data Availability

The TCO-Dataset is openly available on Hugging Face at https://doi.org/10.57967/hf/7744^22^. All data files, including images and metadata, are released under the Creative Commons Attribution 4.0 International (CC-BY 4.0) license. Please refer to the **Data Records** section for detailed information on file structures and formats.

## Code availability

The code used to evaluate the TCO-Dataset is publicly available on GitHub at https://github.com/dosomethingbyme/TCO-Dataset. The repository provides scripts for loading the Markdown question files, TXT answer files, and image files; constructing the multimodal prompts; encoding images for API-based inference; extracting model predictions; and computing accuracy and valid response rate. The repository also provides recommended train/validation/test split files and split metadata for future fine-tuning and domain adaptation studies.
