# Supplementary material for: A multimodal benchmark dataset for evaluating large language models on traditional Chinese opera understanding
Source: Sci Data. 2026 Jul 1;13:1466. doi: 10.1038/s41597-026-07611-7 (PMC13687539; doi:10.1038/s41597-026-07611-7)
Supplement: Supplementary file 1 [file 41597_2026_7611_MOESM1_ESM.docx]

Human Data Submission Checklist

Required for all *Scientific Data* authors describing data that originated from humans (e.g. biological studies, medical data, personal information, survey responses, etc.).

Please note this form is checked at the first decision stage but compliance is ***not assessed by the in-house editorial team in advance of peer review***.

Please ensure that the data sharing method is within our policies (see guidance notes at the end of the document) to avoid future issues and contact the editorial office (scientificdata@nature.com) if you are unsure. Failure to comply may result in delays to publication or rejection of the manuscript. Please:

- read the guidance notes at the end of the document
- answer questions in the checklist and confirm that the relevant information is also included in the main article
- sign the form (only one author is required, signatures may be electronic).
- supply a copy within your manuscript files (file type = ‘Related Manuscript file’)

# CHECKLIST**:**

1) **Please tell us how/if consent was obtained from participants:**

*If data has been collected via multiple routes, please tick all the apply and answer all the related follow-up questions*

☒ Informed consent for participation and data sharing (either at the time of collection, or for future use) was obtained directly from participants and I have described this in the Methods section of my paper.

☐ Check this additional box if a parent, guardian, or other responsible role was required to provide this in cases where the individual cannot reasonably consent themselves, and to confirm this is stated in the Methods section of the paper. **Go to Question 2.**

☐ Patients were not informed or did not provide consent for data sharing (e.g. during routine a medical interaction, non-medical case) but a third party has agreed this may be waived, and I have explained this in the Methods section of the paper. Note this is only allowed for fully anonymised data and not for vulnerable groups. **Go to Question 2.**

☐ Did not interact with or collect data from live patients, but analysed existing samples or material obtained from a biobank, commercial vendor, or other source. Please ensure the source is stated in the Methods section, at a level of detail that would allow others to find and obtain them, and you have compiled with the terms and conditions from the supplier. **Go to Question 7 and skip all other questions**

☐ Did not interact with live patients but downloaded existing human data from an external repository and used to create a secondary dataset. This means the original data owner is responsible for consent, so the requirement is to ensure you have checked and complied with the terms for re-use. Please ensure it is clear in Methods where the data came from, and you have checked and declared (in the paper) your use is within the terms. **Go to Question 7 and skip all other questions**

☐ The data is from another public source or public document (e.g. news articles) but no new personal data is being shared. **Go to Question 7 and skip all other questions*.*** *Please note that social media data is NOT allowed within this exception so please contact the journal prior to submission (scientificdata@nature.com) if you wish to share social media data (e.g. X, Facebook, Reddit, WeChat, Telegram, classified ads, etc.).*

2) **Please tell us what ethics approval has been provided**

☒ Institutional ethics board or IRB (this should match at least one author institution associated with the paper) OR a third party or private IRB (if no such board exists within the authors’ institution(s)) approved the data collection and study. **Complete the box below and go to Question 3.**

*Academic Committee of the School of Automation Science and Engineering, Xi'an Jiaotong University*

*N/A (Ethical approval waived for non-interventional photography)*

*Yes, stated in Methods*

*N/A*

3) **Which overall category of data sharing is being shared?**

*Please note further definitions and risk criteria are given the guidance notes*

☒ **Open data sharing**, meaning an open licence (CC0 or CC-BY – we do not accept -NC licences so please contact the journal prior to submission if you need one), no barriers to download, and reviewers can access the data anonymously. This should be used for non-sensitive data with little or no risk of identification, or (in rarer cases), identifiable data where full, informed consent has been obtained from participants for full open sharing. Please ensure the repository and data link and included in the Data Record section of the paper. **Go to Question 7 and skip all other questions**

☐ **Controlled access**, meaning the data carry a risk of patient/participant re-identification, with or without highly sensitive data fields, and there are tangible risks (e.g. re-identification, personal data disclosure, GDPR) associated with some uses that required controls. Checking this box confirms the limitations are detailed in a Data Usage Agreement (DUA) which all users who require access to the data are requested to sign. Please ensure the details on how to access the data, and the reason for the restrictions, are clearly described in the paper. **Go to Question 4**

*Please note controlled access should not typically be used for non-sensitive data, with no risk of identification, but lacks consent. Please request a risk assessment and consent waiver from your IRB instead.*

4) **What does the data contain?**

Choose all that apply, provide the details, and **Go to Question 5**.

☒ Contains direct identifiers (names, identifiable facial images, biometric data, genomic or transcriptomic data), meaning there is a direct risk of identification/disclosure.

*Identifiable facial images of opera performers (in costume and makeup)*

☐ Contains 3 or more indirect identifiers. We check this as indirect identifiers may still reduce the sampled group to a potentially identifiable cohort (e.g. locations, gender, religion, ethic group, other demographics).

*State which fields*

☐ Contains sensitive or protected fields (e.g. racial or ethnic origin, political opinions, beliefs, union membership, health/diagnosis data, psychological assessments, financial data, criminal convictions, etc). These do not identify the individual directly but present risks of disclosure if the dataset can be de-anonymised

*State which fields*

*Please note that if the data is not identifiable the data may be more suitable for open data sharing, see Q3*

5) **Is the Data Usage Agreement (DUA) available to the editorial staff, and future readers?**

Please tell us where to find the Data Usage Agreement. Note the terms of the DUA will be checked at the first peer review decision (see the guidance notes for our policy). **Go to Question 6**

*The DUA needs to be public, and usually available via the data page / repository. If this is not yet public please supply a copy in the manuscript files as a ‘Related Manuscript file’ and declare that has been done. Otherwise, share a URL for the editorial team to download the DUA.*

6) **Please tell us what practical controls for data access are used and how reviewers can access the data.**

Please explain how reviewers (for peer review) will access the data, and your current or final process for data access upon release of the data and publication of the dataset. **Then Go to Q7**

☐ No practical controls, such as registration/login, authentication, or manual approval. **Please note that there still must be a means for users to read and confirm adherence to the DUA**

- There is no requirement to state this explicitly in the paper if the repository interface is intuitive and users can easily discern how to check the terms and download the data

☐ Automated user registration, verification, and acceptance of the DUA terms without manual screening (e.g. users receive a download link or credential access immediately, providing a valid email address, name, agree to terms, etc)

- Please ensure this is described in the “Usage Notes” section of the paper. **Important:** reviewers need to be sure that and registration process does not reveal their names to you (the authors) and any download links will be available instantaneously after registration and agreeing to the terms. Please create a temporary pdf to state this and upload it to the manuscript files as an ‘Article’ file, appended to the front of the paper, instructing reviewers to use the regular access process.

☐ Manual application or registration process via the repository (beyond basic email validation).

- Please ensure this is described in the “Usage Notes” section of the paper. **Important:** there must be a way for reviewers to bypass these controls or access a sample of the data via direct download, to review the paper. **Reviewers cannot use any data access process that involves a delay in data access or reveals their identities to the research team**. Please create a temporary pdf to explain how to review the data and upload it to the manuscript files as an ‘Article’ file, appended to the front of the paper. E.g. a private link, login, URL to sample, or other method. This needs to provide virtually instantaneous data access to avoid delay.

If you are unable to comply with any of the above requirements, please contact the journal at [scientificdata@nature.com](mailto:scientificdata@nature.com) prior to full submission.

7) **Declaration**

I certify that all the above information is complete and correct.

2026/1/5

Shuo Hou

Typed signature	Date

# Guidance notes

The journal considers two methods of data sharing:

## Option A: fully open data

- Data are fully anonymised or exhibit extremely low risks to re-identification and/or do not contain any sensitive fields
- No controls on use are needed
- Open download without barrier (either direct https download or immediate registration)
- Open licences (CC-BY / CC0, or equivalent). We accept non-commercial clauses (-NC) for secondary datasets using input data shared under those terms, but rarely for other reasons.

## Option B: controlled access data

- Data are not fully anonymised and/or there is risk to participants/patients based on disclosure of any sensitive human data field.
- All users must register to access data, meaning the names and institutions of users are recorded
- A Data Usage Agreement (DUA) or other contract stipulates the limitations on use, and all users sign it

Both options apply to new data shared in public for the first time. Secondary data sharing, where datasets are derived from data that is already available in public databases, is typically not subject to a human data sharing policy but users must ensure they have complied with the terms of the primary owner. The most important aspect to check is whether you have the right to re-distribute the data. Ethics approval for secondary use is generally not required by this journal but may be a requirement of the data owner/platform or your institution.

## Expectations and checks for open or controlled access data (A or B)

**Consent has been obtained from human participants or patients OR an institutional review board (IRB) has waived the requirement**. Waivers are only possible for fully anonymised, non-biometric data where it was not possible to obtain consent at the time of collection and should not be used for children or vulnerable adults.

**An IRB/ethics board has given approval to perform the study AND share the data**

## Expectations and checks for controlled access data only (B)

1. We check whether controls are needed (i.e. why is Option A is not possible)?

Scientific Data follows “As open as possible, as closed as necessary”. This means we start by assuming fully open data sharing as a default and then apply restrictions when there are tangible risks to participants/patients. We anticipate most risks are due to sharing personal/non-anonymised data. All data owners are asked to assess the risks to participants/patients in sharing the data and consider what controls are required to mitigate them. This risk assessment is dependent on the data type (the files) being shared and the presence of either direct identifiers (e.g. genomes, names, faces) or multiple indirect identifiers that reduce the size of a cohort to a potentially identifiable group (e.g. locations, gender, medical characteristics, occupations, etc). Data which are identifiable AND contain sensitive fields (e.g. political or religious beliefs, personal finance data, health data, sexual or gender identity, etc) are considered the most sensitive.

2. We assume controls/mitigations are (mainly) legal protections.

Controlled access mandates users sign a contract (a Data Usage Agreement) promising to abide by the terms. Practical controls and application processes should typically be limited to user registration, identity validation, and ensuring the DUA has been pro-actively signed or agreed to, rather than the submission or assessment of a research proposal. We generally expect all users who can provide verifiable identity information and agree to the terms will be given access to the data and will not be screened on their research question, country, institution, institution type (e.g. academic, government, commercial) or other criteria that does not clearly relate to data protection. In the rare cases where manual assessments are considered useful it should be clear in the paper what questions are being asked, what assessments are being made, and how those checks practically reduce risk.

3. We check the Data Usage Agreement (DUA) **contains** several common terms

While these may vary in wording and specific requirements, most DUAs state users:

- Do not attempt to re-identify participants, either directly or via cross-reference with other sources
- Do not re-distribute the data to any other user not named or signed in the agreement (all users need to obtain the data directly, signing individually or in groups).
- Data should be used for internal research/analysis only, meaning that sharing outputs and derivatives do not breach the “do not redistribute” clause, and there is no external interaction with participants.
- Promise to host/manage the data securely, with sufficient controls, training, etc

Other standard clauses such as: descriptions of limited liability; what to do in the event of a data breach; intellectual property notifications (that the DUA does not add or remove any protections itself); the requirement to cite the dataset in the reference list of any works where it has been used; or any clause included within standard open licences such as CC-BY, are allowed.

An example of an acceptable DUA is the PhysioNet Credentialed Health Data Use Agreement 1.5.0 <https://physionet.org/content/mimiciii/view-dua/1.4/>.

4. We check the Data Usage Agreement (DUA) **does not contain** these commonly disallowed terms

Our general expectation is that DUAs are used to protect human health data, so the journal may seek to remove terms that place restrictions on use but do not look relevant to this aim. If you believe there are any terms that clash with the following, please email us at [scientificdata@nature.com](mailto:scientificdata@nature.com) prior to submission. The following is a list of commonly excluded terms but not an exhaustive list.

- Monetary payment for data access
- Intellectual property claims or other restrictions on allowed derived works that are do not breach non-disclosure of the primary data (e.g. algorithms, results)
- Requirement to share associated publications with the data owner in advance of publication
- Requirements for co-authorship on publications (between the data owner and the secondary user)
- Academic-only / non-commercial use. Restrictions should generally be on usage, not user. “Research only” or “internal research only” may be acceptable if such uses may be explicitly defined and it is clear they allow legitimate, safe, commercial R+D that does not breach other terms
- Accreditation requests beyond citation of the associated dataset and/or publication in the reference list of works that describe their use
- Explicit monetary fines for misuse (above and beyond those codified in existing laws)
- Extensive requirements for research proposal disclosure or application as outlined above. The journal assumes DUA protections are mostly legal, not practical, and trying to assess or judge risk of compliance using research proposals or personal data is not generally viable to reduce it. E.g. via a data access committee, manual assessment, or other ‘data available on request’ process. Where these are used it must be clear what is specifically being checked, and why, and how this reduces risk. Please contact us prior to submission ([scientificdata@nature.com)](mailto:scientificdata@nature.com) if you do want to use a manual/committee/proposal process to explain what practical tests will be applied, with examples or criteria that will be allowed/disallowed.
